# The Drone, the Snake, and the Crystal: Manifesting Potency in 3D Digital Replicas of Living Heritage and Archaeological Places

**DOI:** 10.1007/s11759-022-09460-3

**Published:** 2022-12-02

**Authors:** Stephen Wessels, Sechaba Maape, Benjamin J. Schoville, Jayne Wilkins

**Affiliations:** 1 Department of Archaeology, University of Cape Town, Cape Town, South Africa; 2 School of Architecture and Planning, Wits University, Johannesburg, South Africa; 3 School of Social Science, University of Queensland, St Lucia, QLD Australia; 4 Human Evolution Research Institute, University of Cape Town, Cape Town, South Africa; 5 Australian Research Centre for Human Evolution, School of Environment and Science, Griffith University, Brisbane, QLD Australia

**Keywords:** Virtual Archaeology, Photogrammetry, 3D Visualisation, Multi-vocal

## Abstract

Creating and sharing 3D digital replicas of archaeological sites online has become increasingly common. They are being integrated in excavation workflows, used to foster public engagement with the site, and provide communication and outreach of research, which now happen on digital media platforms. However, there has been little introspection by the community involved in the 3D documentation field, which has resulted in problematic practices. We critique the western paradigm of archaeological visualisation and propose recommendations for inclusive, decolonised visualisations of living heritage and archaeological places. To begin, we define in broad terms what an archaeological site is, and then we describe how these sites have been recorded and represented using the latest technology for digital re-production, namely laser scanning and photogrammetry. Following that we provide a critical analysis of current 3D visualisations of archaeological sites and develop an approach to ensure that the significance, meaning, and *potency* of archaeological and living heritage places are transferred to their digital replicas. Our case study at Ga-Mohana Hill in South Africa then offers practical approaches and methodologies that the fields of cultural heritage documentation and archaeological visualisation can employ to address their recurring issues as identified in the critical analysis. We present an online, interactive 3D digital replica of a living heritage and archaeological place that we believe responds appropriately to its political, cultural, and social context along with communicating its archaeological significance.

## Résumé

La création et le partage de répliques numériques en 3D de sites archéologiques en ligne sont devenus de plus en plus courants. Ils sont intégrés dans des flux de travaux de fouilles, utilisés afin de promouvoir l'intérêt du public pour le site et apporter une communication et une mise à disposition de la recherche, ayant lieu désormais sur les plateformes de médias numériques. Cependant, la communauté impliquée dans le champ de la documentation en 3D a fait preuve de très peu d'introspection, ayant résulté en des pratiques problématiques. Nous critiquons le paradigme occidental de la visualisation archéologique et nous proposons des recommandations pour des visualisations inclusives, décolonisées de lieux archéologiques et du patrimoine vivant. Nous définissons pour commencer selon des termes larges ce qu'est un site archéologique, puis nous décrivons comment ces sites ont été enregistrés et représentés en utilisant la toute dernière technologie de reproduction numérique, à savoir la numérisation par laser et la photogrammétrie. Nous développons ensuite une analyse critique des visualisations actuelles en 3D de sites archéologiques et nous élaborons une approche afin de veiller à ce que la signification, le sens et la puissance des lieux archéologiques et du patrimoine vivant soient transférés à leurs répliques numériques. Notre étude de cas à Ga-Mohana Hill en Afrique du Sud offre des approches et des méthodologies pratiques que les champs de la documentation du patrimoine culturel et de la visualisation archéologique peuvent utiliser pour traiter les difficultés récurrentes identifiées dans l'analyse critique. Nous présentons une réplique numérique en 3D interactive en ligne d'un lieu archéologique et du patrimoine vivant dont nous estimons qu'elle répond de manière appropriée à son contexte politique, culturel et social tout en communiquant sa signification archéologique.

## Resumen

Crear y compartir réplicas digitales en 3D de sitios arqueológicos en línea se ha vuelto cada vez más común. Se están integrando en los flujos de trabajo de excavación y se utilizan para fomentar el compromiso público con el sitio y proporcionar comunicación y divulgación de la investigación, que ahora se realizan en plataformas de medios digitales. Sin embargo, ha habido poca introspección por parte de la comunidad involucrada en el campo de la documentación 3D, lo que ha resultado en prácticas problemáticas. Criticamos el paradigma occidental de visualización arqueológica y proponemos recomendaciones para visualizaciones inclusivas y descolonizadas del patrimonio vivo y los lugares arqueológicos. Para comenzar, definimos en términos generales qué es un sitio arqueológico y luego describimos cómo estos sitios han sido registrados y representados utilizando la última tecnología para la reproducción digital, a saber, el escaneo láser y la fotogrametría. A continuación, proporcionamos un análisis crítico de las visualizaciones 3D actuales de sitios arqueológicos y desarrollamos un enfoque para garantizar que la importancia, el significado y la potencia de los lugares arqueológicos y del patrimonio vivo se transfieran a sus réplicas digitales. Nuestro estudio de caso en Ga-Mohana Hill en Sudáfrica ofrece enfoques y metodologías prácticas que los campos de documentación del patrimonio cultural y visualización arqueológica pueden emplear para abordar sus problemas recurrentes identificados en el análisis crítico. Presentamos una réplica digital en 3D interactiva en línea de un patrimonio vivo y un lugar arqueológico que creemos que responde adecuadamente a su contexto político, cultural y social, además de comunicar su importancia arqueológica.

## Introduction

Archaeological visualisation is an essential practice that facilitates the flow of archaeological information and interpretation from the academic world to the public. The power of visual over textual presentation has been widely embraced and acknowledged by archaeological visualisers (Cochrane & Russell 2007; Opgenhaffen 2021; Watterson 2015) as it allows condensed information to be presented at once, overcomes the limiting linearity of text, overcomes the language barrier, and displays ideas that language may fail to achieve (Cochrane & Russell 2007). Archaeological visualisations, especially when digital, therefore, have a far wider audience than text-based descriptions and have a long history as a powerful tool in communicating archaeological knowledge (Lercari 2017; Opgenhaffen 2021; Perry 2015).

Since archaeology has its origins in a western paradigm (Smith & Wobst 2005), the resulting visualisations usually emanate from a western cartesian perspective. The ontological separation of subject and object, mind and body, and human and nature, have influenced archaeological visualisations. But as anthropology tells us, these separations did not, and still do not, exist for many of the cultures that are being visualised (Casey 1996; Ingold 2000). This paper examines how we can decentre and decolonise the practice of archaeological visualisation, which make use of 3D digital replicas, by first acknowledging the biases in the western gaze of cultural heritage 3D digitisation projects and secondly by deeply engaging with archaeological sites using a phenomenological approach, practising co-production, embracing creativity, and representing and preserving the contemporary values and meanings that are attached to the archaeological place, especially where cultural practices are still happening.

We draw from the objectives of indigenous archaeology in our representation so that “Indigenous values, ideas, expressions, and experiences can be productively incorporated into the discipline” (Colwell-Chanthaphonh et al. 2010, 234). Rather than replacing the western practice of archaeology for indigenous practices we follow the lead of indigenous scholars in seeking to blend the knowledge and epistemologies of indigenous people with the strengths of western archaeological science (Atalay 2006; Londoño 2021; McNiven 2016; Silliman 2009; Watkins 2011). By doing so we aim to bring to the centre important perspectives of living heritage sites, besides the scientific archaeological value and to bring ethics and social justice to a wider and diverse audience.

## Background

### Contextualising Archaeology in South Africa

Human evolution has played a big role in the politics of identity in South Africa. During apartheid South Africa there was a program of misappropriating science to further racist agendas, which included the interpretations of archaeological finds (Esterhuysen 2020). These justifications were in line with the western world which casted African and Asia as other, as primitive, infantile, and not civilised (Athreya & Ackermann 2020). Notions of primitiveness have impacted detrimentally on the identities of affected South Africans to this day (Esterhuysen 2020). The diorama of Khoesan people at Iziko Museum in Cape Town was only recently removed from display in 2013 for the reason of primitivising people and making people the mere objects of scientific curiosity (Athreya & Ackermann 2020).

The fate of Sarah Baartman serves as a profound example of colonial attitudes towards the indigenous people of South Africa. Sarah Baartman was a Khoe woman who was taken to Europe in the early 1800’s to be exhibited as a freak show and “living savage” (Athreya & Ackermann 2020). After her death in 1816 a cast was made of her body that went on display until the 1970s. She was given the name “The Hottentot Venus” which reduced her to an object of sexual curiosity (Athreya & Ackermann 2020).

Another example is Great Zimbabwe, which was a major civilisation of southern Africa. Colonialists refused to believe its African origin and propagated the myth that it was built by Phoenicians, the Queen of Sheba, or “Arabs” (Schmidt & Pikirayi 2018).

These attitudes have had an impact on how indigenous people were treated and made to think of themselves, and many people remain cut-off from their cultural past. Archaeology is still a sensitive topic that needs a new language to re-invent or define itself (Esterhuysen 2020). We, therefore, need to decolonise our science and make space for other voices of people who have historically been the objects of study (Athreya & Ackermann 2020). Archaeological visualisation can play a fundamental role in reshaping how we feel about the past, and how we form our identities in the present.

### Archaeological Places

Tim Ingold formulated the dwelling perspective to critique the cartesian mind–body distinction which seeks to separate humans from their environment. The dwelling perspective “treats the immersion of the organism-person in an environment or lifeworld as an inescapable condition of existence” (2000, 153). When applying Ingold’s dwelling perspective to stone age archaeological sites, we see that these sites are in fact places that people were inescapably immersed and were, therefore, interlocked in the forming of their lifeworld relationships that constituted their identity. There can, therefore, be no separation. In general terms places are where events, narratives, history, memories, and landscape intersect and intertwine (Casey 1996; Thomas & Ross 2013). Archaeological places are continually being given meaning by people with diverse cultural backgrounds in the present, despite them having been created in the past (Thomas & Ross 2013). Therefore, there are multiple understandings of a place and we can see place as “an entanglement of genealogies, a place where past, present, and future collapse” (Harrison 2011, 5).

Places are not fixed, they are continually happening and being reconstituted (Casey 1996). Places gather tangible cultural materials, as found in the archaeological record, but they also gather intangible experiences, histories, thought, and even memory (Casey 1996). “In this way, events and memories are as much a part of a place as are the artifacts and the tangible structures of a site” (Thomas & Ross 2013, 4). In summary, a place is much more than space; they are spiritual, cultural, physical, and social living entities (Casey 1996). Therefore, this paper will be exploring the capacity for 3D models to represent this dynamic and vital nature of places.

### 3D Digital Replicas in Archaeology

Using laser-scanning and photogrammetry to create accurate and detailed 3D digital replicas of archaeological sites has been enthusiastically embraced, and become standard practice, by archaeologists, and the related field of cultural heritage documentation, for site management, structural analysis, site recording, creating virtual access to sites for the public, and visualising reconstructions.

The appeal of these interactive 3D models over traditional representations of the site such as 2D plans, sketches, and photographs is that they are not only able to record and display high-resolution spatial detail of the site, but they also enable the viewer to move through/manipulate the environment.

As Ingold (2000) notes, we perceive the world by moving through it. Therefore, enabling movement and manipulation of space creates a much more vivid perception of the site, albeit visual. We can better perceive its physical dimensions and colour than from frozen perspectives offered by traditional sketches, 2D plans, or photographs as the manipulation of the 3D models allows the “observation of and interaction with the minute details of objects’ geometry, colour, and texture, thus, evoking a contact with their physical counterparts” (Cardozo & Papadopoulos 2021, 521).

When these 3D models are uploaded online to publicly accessible platforms, the models become visualisations representing the site, and they enter a whole new (digital) world of possibilities, providing access to the sites as never before, and representing the site to a larger audience. Since the models are created in a digital environment, there are opportunities to insert interpretative devices that cannot be created at the physical site, but that would enhance the visitors’ experience of the place. When the models are given interactive functionality, they also allow engagement with the representation, giving agency to the viewer. The models can additionally be accompanied by annotations and multimodal contextualisation, which provides insights into objects context and biographies (Cardozo & Papadopoulos 2021). As Piccialli and Chianese (2017, 187) note “Innovative applications and services can shorten the distance between cultural spaces, such as museums, art exhibition, historical center and archeological parks, and citizens”. 3D digital replicas have the ability to provide powerful modes of visualisation, perception, shared access, and engagement. They also have the potential to function as a valuable repository of tangible and intangible cultural knowledge. According to Stuart Jeffrey (2015), having these digital tools at our disposal should herald a Golden Age of archaeological visualisation; however, we have not yet fulfilled this potential.

#### The Creators of Digital Replicas

3D digitisation initiatives that focus on the accurate and objective production of 3D models as digital replicas of the physical site have produced databases of 3D models of sites which they host online for the public to view (for example Cyark, Zamani Project, Global Digital Heritage). Some of their aims include creating records of the physical sites for future generations as a response to the threat of natural and man-made destruction, as well as creating awareness for the site and creating materials for education and tourism (Rüther 2002; Rüther et al. 2014, 2009; Rüther et al. 2012; Wessels et al. 2014). University groups and enthusiasts are also drawn to cultural sites as they offer interesting structures to capture and reproduce. In the last two decades, 3D documentation technology has improved and become cheaper, which has resulted in its usage dramatically increasing. Laser scanning and photogrammetry dominate spatial capture methods and are very well suited for the purpose of recording heritage sites (Rüther et al. 2012). But we need to ask what is exactly being recorded for preservation?

#### Viewing the Models

In general, the public are limited to 3D models that they can access for free online of sites with the minimum technological means (low-bandwidth, minimum device processing power). As the Covid-19 pandemic closed borders and classrooms, many educators scrambled for digital models of objects and places that could be safe substitutes for in-person experiences. There are many platforms that host interactive 3D models of archaeological and heritage sites (see Champion & Rahaman 2020 for a full list and comparison). By far the most popular platform is Sketchfab, with over 200,000 cultural heritage models hosted online (Champion & Rahaman 2020). Sketchfab is a platform that encourages cultural institutions to upload their 3D models of sites and objects, by offering them incentives and actively encouraging open access by allowing models to be downloaded, under a range of creative commons licencing options (Flynn 2020). It is these online models that constitute the vast majority of 3D visualisation of archaeological sites, and it is subsequently these models that form the online digital presence of these places, which we discuss in more detail later. The following critical analysis is, therefore, based on these models.

### Critical Analysis of 3D Digital Replicas

From a philosophical perspective using laser scanning and photogrammetry to document sites parallels the way “European nations carved up Africa, using Cartesian tools of rationalisation to give it new meaning grounded in sinister dispossession” (Maape 2021, 64). The maps created by colonial surveyors presented places as empty of people and hence commodifiable. Essentially all meaning was stripped from a place during its mapping and subsequent political processes. It was these processes of de-meaning that have alienated people in South Africa today, where their connection to their past has often been severed (Maape 2021). There is a threat of these same actions being repeated, regardless of whether it is intentional or not, in the documentation and visualisation of archaeological and living heritage sites.

Some argue that interactive online 3D models may result in de-humanising places and are inauthentic, sterile, and unengaging representations of the original (Huggett 2020; Jeffrey 2018; Tan & Rahaman 2009; Watterson 2015). Their primary grievance is that the creators of these models focus on representing a space, with little attention and effort directed towards giving that space meaning that would turn it into a culturally meaningful place (Ibrahim et al. 2015; Jeffrey 2015; Pujol 2017; Thomas & Ross 2013).

There exists a danger of technological fetishism that seems to pervade these projects, where the quest for ever more realistic, precise, and accurate models is an end in itself (Huggett 2004; Jeffrey 2015). As Stuart Jeffrey (Jeffrey 2015, 149) notes “digital representations of the past continue to struggle to overcome the perception that they are either purely scientific tools for analysis and management or flashy and unnecessary demonstrations of technological prowess offering no real insight into or connection with the past”. As Priscilla Ulguim (Ulguim 2018, 3) also argues, “how we share 3D models and what we communicate about them are just as important as the technologies we use to create them”. An additional critique is that only the visual aspect of the site is being recorded by documentation projects, with our other senses currently being ignored (Eve 2018). While the objectives of these institutions, teams, and individuals may clearly be to record space only, we draw attention to the implications of this technology-focused documentation approach (which we call the objective approach) for the subsequent perceptions the public generates about the site when viewing visualisations of their resulting 3D models in an online public setting.

Critics of the objective approach point out that the resulting models are actually not objective representations as they are intended and claimed to be. A photograph of an archaeological site is subjective because it is framed from a particular perspective that is not explicitly described (Morgan 2012; Reilly et al. 2021; Smith & Blundell 2004; Watterson 2015). Similarly, 3D models have their own subjectivity because decisions are actively made during the spatial data-capturing process in regards to what the boundaries of the model should be, what the resolution should be and what details should be focused on, to name a few (Cameron 2007; Jeffrey 2015, 2018).

The subsequent processing of the captured data to create the resulting 3D digital replica is also not objective as many subjective decisions are made during the process. Cleaning the model involves removing vegetation, and evidence of people, such as furniture, litter, domestic animals and fire places that are unavoidably captured during the field work but that “detract” from the site. These are all subjective decisions. Correcting errors in the model resulting from the meshing and texturing processes, as well as final optimisation of meshing and texture resolutions are also subjective decisions that people perform. But sites are presented as objective models representing a record of the place as it is now, minus any physical artefact not deemed to belong to the site.

Cleaning, correcting, and optimisation are somewhat unavoidable, since visualisations rely on aesthetics, but we need to be aware of the implications of the process. The 3D visualisations have, thus, been sanitised during the multiple subjective decision-making processes, in the quest to produce an aesthetically appealing model. The results of these cleaning and correcting processes show sites as being devoid of humanity as people are absent along with the evidence of their presence. This in turn creates distance between us and the representation of the place (Bendicho 2013; Jeffrey 2018; Watterson 2015). The concept of paradata, which is a record of the decisions taken to produce a 3D model, has, therefore, been introduced to address this issue and forms one of the Seville principles, an international initiative to provide ethical guidelines for tangible archaeological and heritage visualisations (Bendicho 2013; Reilly et al. 2021). Paradata also create authenticity of digital replicas by providing provenance and data transparency (Amico et al. 2018).

Another criticism is that the site is presented as static, frozen in one moment in time. Indeed, the trees do not sway in the wind, if they have not been removed from the model during the processing stage. However, places are not fixed, they happen (Casey 1996). Sites are living entities where meaning is continuously changing as people evolve. Modern site management practice emphasises a site as being alive and showing a continuation as being important (Thomas & Ross 2013). Just as a video is more engaging than a photograph, an enlivened 3D model is more engaging than one frozen in time.

A side effect of the so-called objective 3D replica is that any creativity imparted onto the model is shunned as being subjective. There is no room in the so-called objective approach to introduce elements that are not firmly recorded by the scientifically proven capturing technology. Alice Watterson (2014) argues that archaeological visualisers need to accept and embrace creativity to create more engaging user experiences, and also document their creative processes.

#### Living Heritage Landscape

Living heritage landscapes where contemporary people are performing cultural activities at places which have past archaeological value as well as present value are appearing online, in digital form. Places, where according to locally practised culture, certain people should not enter, are becoming virtually accessible. Examples of this includes Uluru in Australia where many freely accessible models of this site exist on Sketchfab, with some of them being available for purchase. The ethics behind this practice are important to consider and deal with. However, to some extent we must acknowledge the inevitable situation that the whole earth is being digitised. Google earth allows us to view almost anywhere and the resolution of their models is ever increasing. While these ethical battles about virtual access should no doubt be fought, we need to also deal with the consequences. Living heritage places, where multiple meanings co-exist, are exposed online, but is it the way that sites are presented, and their context, that is the issue we wish to address. We see that each site is unique, sites have their own independent realities, and so any such approach to representing them needs to be flexible, and be tolerant and accommodating of differing views (Ross & Thomas 2013).

### Digital Presence

We argue that these online 3D models as critiqued above form an archaeological site’s digital presence. They are virtual worlds representing the real place and are essentially the continuity of the physical place into the digital realm. Digital replicas of sites have their own network of meaning and are not just snapshots or echoes of their real-world counterpart (Morgan 2012). Our future points to an ever more digital existence, and so these models will become far more than visual records of a site at a moment in time, they are responsible for representing a site in its digital form. The success that they do that will be in how people can engage with the site, learn about archaeological places, and create their own meanings about it. Most young people access heritage through digital means today. The public, therefore, needs to be presented with an authentic experience when visiting these 3D models, but currently the visualisations are not faithfully representing the place, only the empty space. Encountering these models, therefore, creates a skewed sense of the place, its importance, and its history, while also reducing the potential for meaningful connections to the place.

It could be argued that it is not within the scope of these documentation projects to preserve the culture or intangible aspect of the places, as that is not their expertise. Whether or not this is valid does not change the fact that by uploading models online they are constituting the place’s digital presence by facilitating access to the model. We argue that this action comes with responsibility.

We must, therefore, take all the above into account and deal with the many challenges involved when producing visualisations of archaeological places if we want to create a visualisation that does not devalue places but rather preserves their potency.

## Method

### Conceptualising the Visualisation

From the critical analysis above it can be seen that 3D digital replicas are currently focused on replicating space, and not place, which has resulted in the 3D visualisations lacking in engagement, meaning-making and significance. As a response to this observation, an approach was developed that has the objective to replicate archaeological and living heritage sites as places and not spaces.

The following three aspects were identified as being essential to constitute 3D replicas as meaningful places. (1) Agency—having agency is the ability to act upon people to give and receive meaning. (2) Multi-vocality—being multi-vocal seeks to accommodate different perspectives, past and present, of a place and supports a decolonised, de-centred, and inclusive representation of a place that is relevant and appropriate today. (3) Proximity—the significance of an archaeological and heritage site is its ability to provide proximity to the people who used the place in the past and who use it in the present. Digital replicas must, therefore, seek to provide a faithful experience of proximity to the physical place and the people who use it. We aim to implement these three aspects using the objectives below.

**Agency**

- Show the place as a living entity
- Show the place is not static and has a biography from its distant past to the present
- Re-humanise the 3D model
- Enable engagement with the place via its 3D replica

**Multi-vocal**

- Respect the site and the current users of the place
- Represent the multiple perspectives of the site
- Bring to the centre other uses, past and present, of the site besides its scientific significance
- Create a decolonised visualisation of the place

**Proximity**

- 3D model of the site and artefacts must be good quality (visually appealing and accurate) to provoke the feeling that the virtual visitor is there at the site
- Facilitate inclusive access to the site
- Mediate the experience to preserve the atmosphere of the place, and communicate its narrative appropriately
- 3D model must be given authority and authenticity

### Ga-Mohana Hill Case Study

We embarked on a case study approach that attempts to overcome the many challenges outlined in the critical analysis, and to show how the above aspects can be practically implemented.

Ga-Mohana Hill and rock shelters in the Northern Cape province of South Africa was chosen as our case study location. We present a visualisation of Ga-Mohana Hill as an online interactive 3D model residing on Sketchfab.

Ga-Mohana possesses significant archaeological value as a record of early human origins and evolution (Wilkins et al. 2020, 2021). In the recent past it was a place of Khoesan occupation and it holds significant cultural value as a place utilised by members of nearby communities for prayer and ritual (Maape 2021). Ga-Mohana Hill was, therefore, chosen as a case study for this research as it functions as an intersection point of varied belief systems, some of which are diametrically opposed to each other, but are all important and intrinsically linked to the place. These systems of belief include spiritual and ritual meaning, as well as scientific practices, which generally eschew matters of the spirit. We want to decolonise representations and show that forms of value can co-exist. If successful, we will have transferred the potency of the place to its digital 3D form and we will have turned 3D digital space into meaningful place.

#### Description of Ga-Mohana

The physical site of Ga-Mohana Hill, also known as Kurumankop, is situated 12 km northwest of Kuruman at the edge of the southern Kalahari Basin. The hill tops out at 150–180 m above the surrounding landscape. Two significant rock shelters facing northwest (Ga-Mohana Hill North Rockshelter) and southeast (Ga-Mohana Hill South Rockshelter) sit at opposite sides of the hill. The region is characterised as semi-arid savannah. The hill is located on common/tribal land owned by the Baga Motlhware Traditional Council.

#### Co-production

This case study project includes four authors from three different disciplines and backgrounds the goal of which is to intentionally bring different perspectives to the visualisation. SW is a geomatician with a background in digital 3D re-production of heritage sites and objects. SM is an architect who brings philosophical insight into indigenous knowledge and representation. SM grew up in Kuruman where he is a member of the local community and has done extensive research into ritual practices in the area. JW and BS are archaeologists who lead excavations at the Ga-Mohana Hill North and South shelters.

#### Contemporary Significance of Ga-Mohana

For some local residents of Kuruman and the surrounding area Ga-Mohana Hill is regarded as a spiritual and cultural place and is used for prayer and ritual (Maape 2016). Myths of the Great Snake, Noga ya Metsi, are associated with the hill, and some people in the local community regard the place with mystery, secrecy, and fear. Church groups, such as the Faith Mission Apostolic Church of Zion, use the hill and shelters for prayer sessions and its members will often spend the night in the shelters in spiritual communion. Ga-Mohana Hill plays an important role in maintaining the intangible heritage that is linked to Kuruman and surrounding areas.

SM grew up in the local community of Kuruman, and has conducted extensive research into the contemporary uses, values, and meanings of certain sites of archaeological importance around Kuruman. He describes Ga-Mohana Hill from the perspective of the local community, to which he belongs, in his article ‘*Drawing creepy places: Representing liminal ritual spaces of Kuruman—South Africa’* (Maape 2021). In the article he describes that some of the local community prescribe to an animistic worldview where “water, earth, sky, rock shelters, and caves are full of vitality, animated by the flux that defines the landscape” (Maape 2021, 55). To connect to the vitality of the landscape local people engage particular spaces as a way to adapt to change and make meaning.

Local stories of Kuruman describe a mythical snake which is “the cause of natural forces like whirlwinds, responsible for environmental calamities, lost people, and other tragic situations” (Maape 2021, 55). The snake is said to abduct people, forcing them to experience traumatic, life altering events. The snake is able to shape-shift to fool its victims, and then steal their agency. “Change is forced on those who meet and are abducted by the snake, becoming a kind of grace, invoked through ritual as, ultimately, a death of the self” (Maape 2021, 55). Because of the potency present at these shelters, which manifests from the narratives described above, these shelters are sometimes used for initiation ceremonies, especially for boys.

#### Community Use and Engagement with Ga-Mohana

Ga-Mohana is situated in the general Kuruman community, which is diverse. This is because Kuruman attracts people from the wider region, drawing people from the Southern African region including Namibia and Botswana who come for work at the mines. There are several mines located near Kuruman, including in Kathu, Hotazel, and Sishen. These active mines support large numbers of families who are both local as well as migrant.

The local community in Kuruman, although it is difficult to discern who the locals are, and has become an ongoing point of contention, could be identified as being predominantly Tswana, and of that having the two dominant clans being the Batlharo and the Batlhaping. These two groups, particularly the Batlhaping are historically represented in written records, and currently are the reigning chiefdom in the area, and have been reigning at least as far back as the 1700’s (Shillington 2011). However, there is little to no data to indicate precisely what the cultural demographic make-up of the region is. What is observable however, is that there are individuals who are also Tswana, who migrate from the wider region and live nearer to the local business district, as well as near places of work like the mines or agricultural farms (Maape 2016). Again, it is difficult to determine which of these people are local because the Batlhaping clan in particular stretches perhaps as far as a 300 km radius, so although people may identify this way, they are not necessarily locals.

The two consequences of the above is that the context in which Ga-Mohana is located is diverse, firstly, resulting in a variety of uses of Ga-Mohana, and secondly creating a situation where there is a lack of a homogenous interest group that is advocating for the preservation of the site.

The site is officially administered by the Baga Motlhware Traditional Council; however, there is very little formal restriction and control over the use and access of the site. We have observed several different visitors to the site, who all use it in a variety of ways, and by no means all perceive it to have one meaning. For instance, there have been a variety of ritual uses of the site, which is observable by the presence of various ritual symbols including crosses, written names of churches, candles, presence of multiple fires, knives with blood and feathers and water bottles. This is also corroborated by various individuals who claim to have performed rituals, prayer, and other spiritual activities at the site, all from diverse groups.

Other activities that have been observed and recorded at the site are teenage initiation, visits by traditional healers both local and those visiting from outside, local church groups from different Christian denominations including Pentecostal churches, Catholic and African Independent Churches. Some of these, particularly the latter, aim to maintain secrecy of the place, and on some occasions prohibit access to the site, but this is done largely through mythical and spiritual restriction as opposed to physical and legal rights. Sometimes the place is visited by individuals, sometimes by groups, herders, and sometimes by community members searching for firewood. More recent have been hikes to the site organised by local youth who are attempting to promote tourism in the area (The Workshop ko Kasi 2017).

The second consequence is that the site does not have a homogenous interest group, that is formed out of a homogenous community who share the same values, of which are embodied in the way the site is used and managed. Currently, as mentioned, the site is managed by the local traditional council, however, the council does not exert control over the activities at Ga-Mohana or enforce any restrictions. As a result of the lack of management at the site, and a general lack of a single interest or advocacy group, the site is vulnerable, particularly when for instance archaeologists who in general will have access to the site, engage in their activities there. In (Maape 2021), the argument made was that the value of sites like Ga-Mohana is their intangible heritage, which through new forms of engagement, may add value to the community, and mitigate further loss of cultural heritage.

#### Archaeology at Ga-Mohana

Ga-Mohana Hill’s archaeological potential has been known by the archaeological community for some time due to the presence of painted rock art on the rockshelter walls, and engravings near the base of the hill (Beaumont & Morris 1990). The first systematic excavation programme was initiated in 2016 by a University of Cape Town led team of international researchers, which revealed intact Middle Stone Age (MSA) and Later Stone Age (LSA) archaeological horizons relevant to understanding the origins and evolution of our species, *Homo sapiens* (Wilkins et al. 2020). The oldest MSA occupation of Ga-Mohana Hill North Rockshelter was dated by optically stimulated luminescence to 105,000 years ago (Wilkins et al. 2021). Archaeological artefacts from the site indicate an early origin for important innovative behaviours in the southern Kalahari Basin, and challenges the dominant narrative that these kinds of innovative behaviours appear first at coastal sites and as adaptations to coastal and near-coastal environments. Of particular relevance are several unmodified calcite crystals. They do not originate in the shelter, with the closest potential source of crystals being roughly three km away. This indicates that the crystals were intentionally brought to the shelter. The crystals have no interpretable functional use, and the most probable explanation for their occurrence in the MSA layer is that they served a ritual purpose as has been suggested at other archaeological sites in southern Africa (Chazen & Horwitz 2009). Analyses of the tufa deposits on the hill have revealed that there was a much wetter climate at the time of 105,000 years ago, which is in contrast with today’s dryer climatic conditions. Ostrich eggshell fragments from the MSA level may represent the remains of water storage containers. Together, the archaeological evidence from Ga-Mohana Hill reveals a long history of the technologically and socially complex innovations of our early human ancestors. The results of this archaeological work received international media attention (Wilkins & Maape 2021), and thus, the archaeology of Ga-Mohana Hill is a recent, but major actor on the global archaeological stage.

The archaeological methods employed at Ga-Mohana Hill evolved over time in response to its contemporary cultural significance. To recognise and respect the site's importance the archaeology team employed a number of approaches to minimise their impact on the landscape and the people who use it. The team conducting archaeological excavations at the site have minimised any lasting effects of their excavations. This they have done by completely backfilling and camouflaging the excavations after each field season, positioning control points as inconspicuous as possible, not erecting any permanent structures such as signage, and not altering any paths to the site (Wilkins et al. 2020).

#### Two Value Systems

We, therefore, have in Ga-Mohana a living heritage landscape that is rich in scientific value, where archaeology has revealed important discoveries according to established academic methods. We also have a place rich in current cultural value tied to the past: mythical snakes feature prominently in the ethnography and ritual of the Khoesan, the first people of South Africa, (Orpen 1874; Sullivan & Low 2014, Hoff 1997). There is evidence showing the presence of the Khoesan at Ga-Mohana in the form of rock art and engravings. Ritual is deeply embedded in African traditional culture, and here we have a place showing a connection between past and present peoples: usage of the place for ritual purposes. The two ontological systems of western and indigenous knowledge are firmly represented at the site and we wish to represent these diverse interests using our case study 3D visualisation.

### Creating the 3D Model of Ga-Mohana Hill and Shelters

The following sections describe the process we undertook for the creation of the 3D model for the visualisation. We have included a description of the various decision-making processes as a way to be transparent and acknowledge that the 3D model is not a purely objective representation of the site, but a model that is inherent with subjective decisions. This description effectively forms the paradata of the model that the Seville principles call for (Bendicho 2013). Following that we describe the decision-making process we undertook for mediating the visualisation.

The 3D model of Ga-Mohana Hill was created using photogrammetric imagery and differential GPS measurements. The chosen area to be recorded was 1.5 km × 1.8 km. This large area was chosen to capture the entire hill, and an appropriate portion of the surrounding landscape. We wanted to make sure the two shelters, where most of the human activity we wanted to represent, was well contextualised within the hill and landscape.

#### Data Capture Policy Employed

SM approached the communal land owner, via a letter, requesting permission to record the site. An appropriate fieldwork plan was created taking into account the sensitive nature of the site. The plan involved the following: If we encountered anyone at the site engaged in worship/ritual we would not disturb them at all, and would wait until they had concluded before continuing with our work. A local tourist guide was hired to accompany the data capture team at all times. The guide helped with organising logistics, transporting equipment, and explaining to people on site in their local language what we were doing.

#### Data Capture Fieldwork

The spatial data of Ga-Mohana Hill were captured over four days in February 2021. Firstly, a GPS survey was conducted over the entire designated area to capture control points used to create the 3D model of the site. The control points are necessary to create a correctly scaled 3D model of the hill, as well as to tie into the existing co-ordinates for all spatial data captured on site.

The generated model is tied to the UTM coordinate system of control points established for archaeological excavations at Ga-Mohana Hill in 2016 and 2017. These points were used for the excavations and were marked inconspicuously as either small drilled divots, or painted points. These points were fixed to the 34S UTM grid and South Africa 2010 Geoid. The 2021 GPS survey captured these previous points as well as points covering the entirety of the hillside. Most of these landscape points were of natural features such as well-defined corners of rocks that could be identified from the aerial drone photographs. By integrating previous mapping with the 3D model, the location of mapped artefacts during excavation aligns with the photogrammetry modelling.

Next a drone orthophoto flight was conducted to capture the entire hill. The drone flew a grid pattern with 75% overlap between images with a ground surface density of between 1 and 4 cm depending on the height the image was taken above the landscape. Following this, the drone was used to capture high-resolution SfM images of the two shelters with the camera tilted from nadir to more oblique angles. Finally, a terrestrial photogrammetry survey was done inside the two shelters to record high-resolution detail in the shelters. The images were captured following a 60% overlap procedure and captured the control points at the two shelters.

#### Data Processing and Optimisation

All photogrammetric images were pre-processed in Lightroom software to balance tone, colour, and white balance and convert RAW images files to jpg. All captured GPS points were corrected to the already established excavation reference system.

Next the software Reality Capture was used to align all images, which included identifying and marking the control points in each relevant image. Once satisfactory alignment had been achieved, a mesh was generated and then textured.

#### Decisions During Model Cleaning

This model was optimised for viewing online using the platform Sketchfab. Since we especially wanted the model to be freely accessible to all people with respect to bandwidth considerations, the model was optimised to create the lowest feasible model possible that ensured a balance between detail and file size.

To achieve this the model was split up into separate parts, and these parts were optimised in terms of their triangle count, and texture resolution. The model was, thus, split into shelter (north and south), surrounding area of shelter (north and south), hill-top, and landscape.

### Recording Archaeological Artefacts

The most relevant archaeological artefacts for this model were identified as coming from the dark brown silty roofspall unit (DBSR) in excavation Area A of Ga-Mohana Hill North Rockshelter, which dates to 105,000 years ago (Wilkins et al. 2021). The chosen artefacts consisted of three calcite crystals, four stone tools and five ostrich eggshell fragments.

The artefacts were photogrammetrically captured using a turntable and lightbox.

Images were then pre-processed in Lightroom to correct for colour and white balance and 3D reconstructions were created in Reality Capture with the models and textures cleaned up in Blender.

The ostrich eggshell fragments were not photo-scanned as they were too small, but modelled in Blender software.

### Creating the 3D Interactive Visualisation

Our first creative decision was to visually contextualise Ga-Mohana. We used as a backdrop to the 3D model an unmistakable photograph of the Milky Way (photo credit: NASA) which encourages thoughts about human’s unique place in the universe, being alive and conscious to experience it, as well as to think about the different cosmologies that are represented at the site.

Our initial plan was to incorporate into the model the drawings SM had done for his project “Drawing Creepy places” (Maape 2021). These drawings were created in a self-reflexive process to explore and make visual his feelings of the shelters and map these onto the landscape.

This idea was tested, but abandoned in favour of a more subtle approach. We did not want to impose too much onto the landscape, as we were aiming to be multi-vocal in our approach, and felt that by imposing one view we were emulating the colonial project. We wanted the site to speak for itself, and we felt the drawings would interfere with people’s perception of the place as they navigated around the 3D model. We wanted to encourage people to generate their own feelings as they explored the site.

We decided on the metaphor of light and darkness as the general theme of the visualisation. The colonial project sought to illuminate the dark continent of Africa, to lift the veil of its mysteries and transform its people to the so-called civilised culture of the West. In contrast to this idea, many indigenous rituals make explicit use of darkness as a means to incite a particular feeling. Fear, secrecy, and mystery are all associated with the dark. Illuminating the dark, therefore, strips the potency from these ritual places (Maape 2021).

We, therefore, decided to embrace the darkness, and carefully arranged our lighting of the site to keep certain areas in shadow. We also wanted to acknowledge the role and presence of the Great Snake in the site. As SM has pointed out “Any representation of sites associated with the snake must therefore address the difference between objective and animistic space and find other ways to represent their nature as being the snake” (Maape 2021, 57). The snake is said to inhabit the cave, but it also represents the cave, the universe, and is a contingent force that is responsible for life’s seemingly random hardships and miseries. It, therefore, acts as a humbling agent, an ego diminisher, forcing people to be aware that they are not in absolute control of their fate.

To represent this notion, we again employed the metaphor of light and dark. We attached a light source to the viewer, but pointed it at right angles to the viewer's forward view. This device then creates an unpredictable result when rotating in the 3D environment. The viewer gets the feeling that they are not in direct control of what is illuminated and what is left in shadow, but also creates a means for the cave to be lit up, just maybe not when it is intended. This dynamic lighting, therefore, created agency for the shelter and represents the snake. It gives the site a feeling of being alive by casting shadows haphazardly around the shelter.

**Figure 1:**
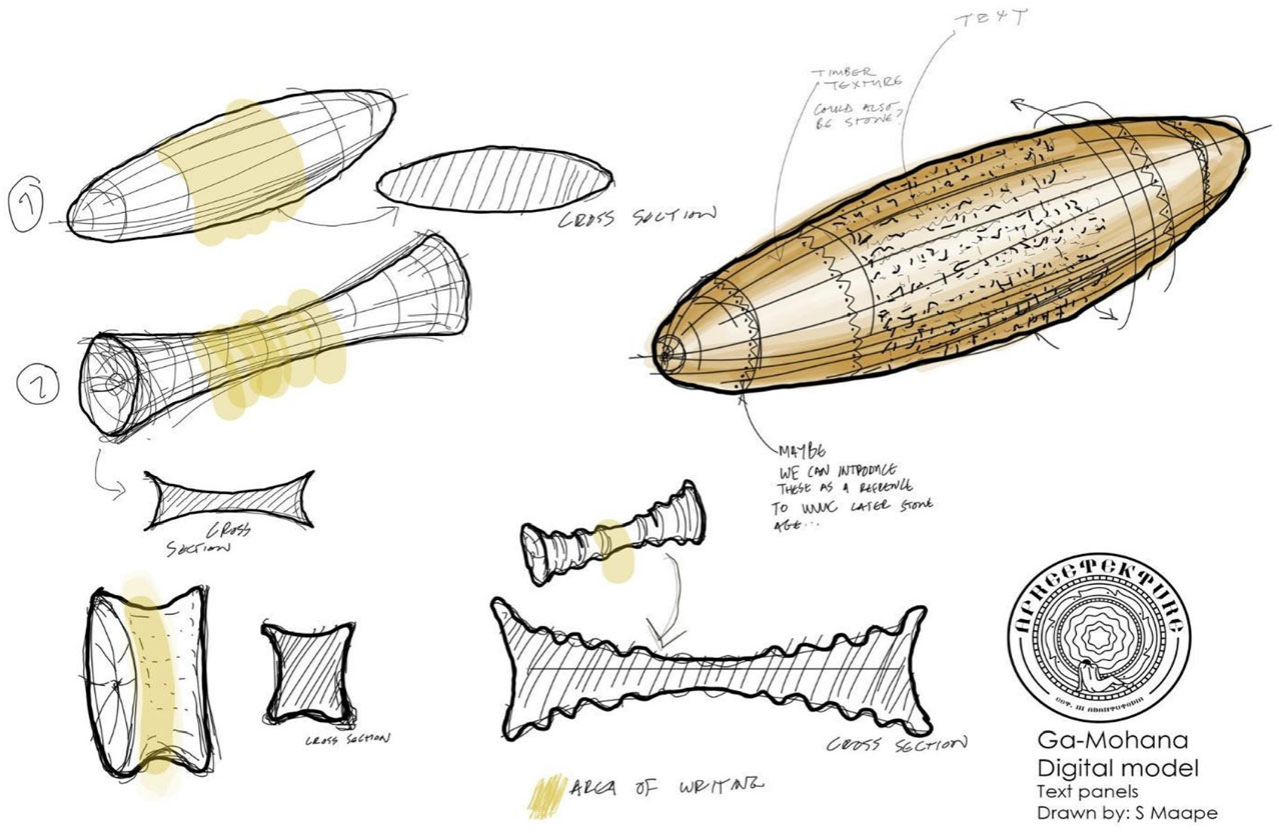
Conceptual drawing by SM of the rotating signboards

Next, we developed the flow of the movement the viewer experiences while navigating through the site. The flow is created by annotations that are built into the functionality of Sketchfab. Using the annotations, the viewer can easily move between the points of interest that we created around Ga-Mohana. The points of interest are numbered to encourage a sequential movement, but this is not enforced. The viewer is given free control and agency of how to view the site. However, each point of interest is carefully curated with one aspect of the curation being to define the focus point of each point of interest, which was achieved by choosing a particular point of rotation for each annotation. The rotation point will either remain at the centre of the view, such as when we wish to focus attention on particular objects such as the archaeological artefacts, or the point of rotation is located at the viewer's location, which results in sweeping views where nothing is fixed or focused on (Figure  1).

Realising there is no way to avoid using text in the visualisation to provide the information we wanted to provide we created unique, rotating sign boards. This was partly to create movement that captures the viewer's eye, who will be drawn to read the text. It also serves as a device to display text, but not all at once, which would otherwise block out the view of the model. The unique designs on the rotating text objects were inspired by archaeological artefacts discovered in South Africa at Diepkloof rock shelter (Texier et al. 2013; Wessels 2020) to intentionally highlight African patterning and visual style. The rotating objects were placed at each point of interest. The rotating sign boards mimic a sense of authority and authenticity in the same manner of sign displays at museums.

### Website

To contextualise the 3D model of Ga-Mohana Hill and prepare virtual visitors for entering the 3D model, we created a website to house the model. The website begins with a video introduction to the 3D model which briefly explains that the site has significance and meaning. When entering cultural sites in the real world, there exist certain rules and obligations that the visitors must observe to preserve the integrity of the place. We wanted viewers to be aware of the site's significance before entering so that they could be in the appropriate mind-frame to absorb and respect the meaning of the place. The video also shows the visitor how to navigate the model.

The website goes on to describe the context of Ga-Mohana and the Great Snake that is said to inhabit its rock shelters. Other information included in the website explains the purpose of the 3D model, how the 3D model was created, and shows some 2D visual interpretations of Ga-Mohana based on interpretations of historic life at Ga-Mohana.

## Results

### Website and 3D Model

The 3D model visualisation of Ga-Mohana Hill can be accessed via the Ga-Mohana website at https://rockartportal.org/Ga-Mohana.html.

### Annotated Positions

The following describes each annotation of the 3D visualisation, and explains the decisions taken for their curation.

**Figure 2:**
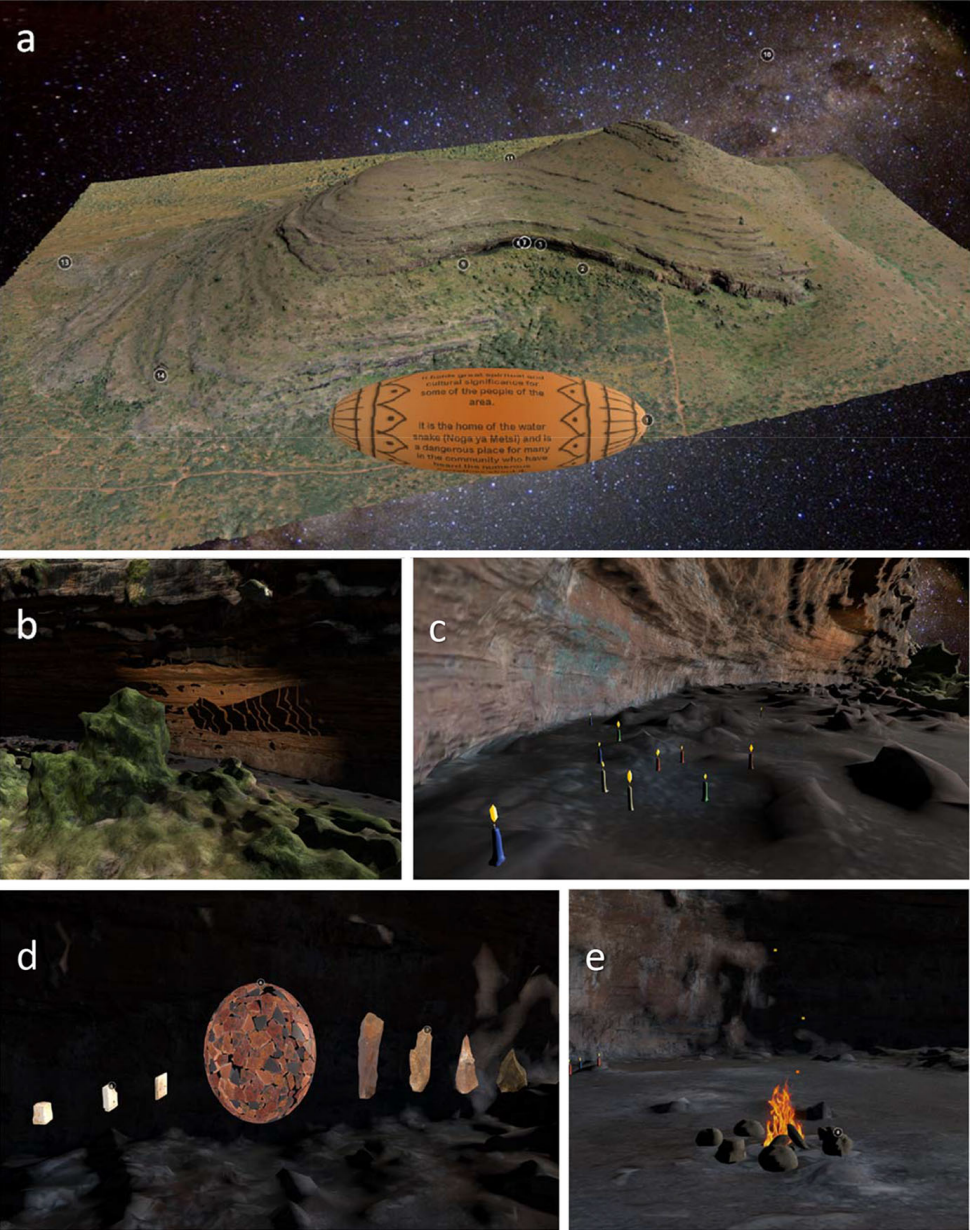

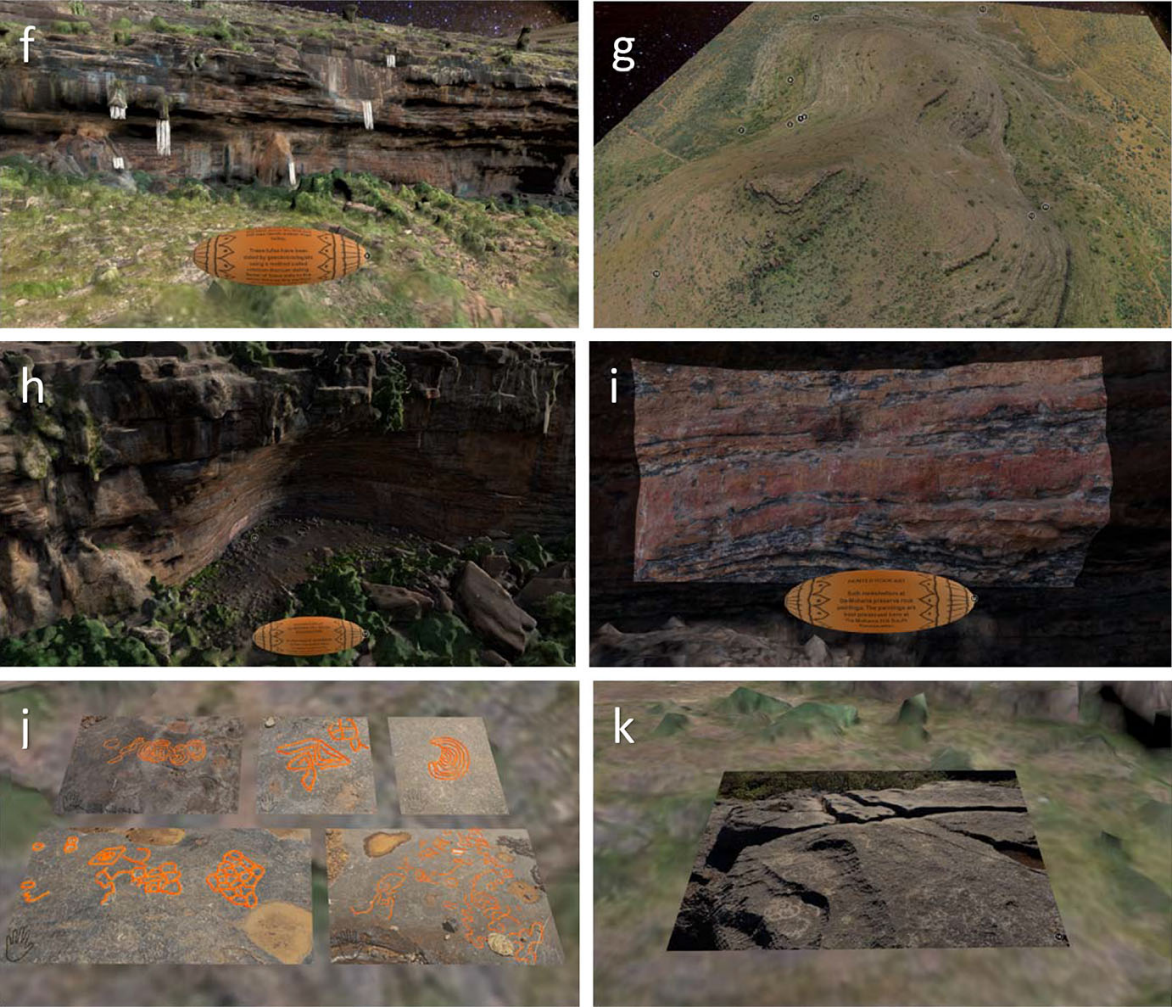
Screenshots of the Ga-Mohana Hill 3D model visualisation from **a** Annotation 1, an overview of the hill; **b** Annotation 2, the shadow of the snake in the shelter; **c** Annotation 3, the candles in the shelter; **d** Annotation 4, 5, 6 and 7, the artefacts found during the archaeological excavation of GHN; **e** Annotation 8, the fireplace; **f** Annotation 9, the tufas; **g** Annotation 10, the entirety of the hill; **h** Annotation 11, Ga-Mohana South Shelter; **i** Annotation 12, the painted rock art in the shelter; **j** Annotation 13, the engravings; **k** Annotation 14, the rock gong

*Annotation 1 *places the viewer at a location allowing a view of the entire model of Ga-Mohana Hill. Here, we placed a rotating object with text that serves to make people aware that they are about to enter a culturally significant place. We also wanted to warn members of the local community that they are about to enter the snake’s shelter. If they form part of the community who practice ritual initiation at the site, and therefore, are forbidden from entering the place without being initiated, then they must be aware and potentially abstain from entering the digital model (Figure 2a).

*Annotation 2* represents the snake. Its physical manifestation is not visualised as the snake does not necessarily inhabit the physical realm. Instead, we chose to represent the snake in the form of its shadow, as it passes over the shelter, in front of a fire—the primaeval tool of life. The site is, thus, alive and houses otherworldly beings. The shadow of the snake is also adorned by patterns, referring to rituals and cycles. The snake is rendered in such a way that is kept ambivalent and mysterious, one never sees its entire body, similar to the way one never sees the model fully illuminated. The snake emerges from the shadows and returns to the shadows the same way it exists in the mythologies and narratives about it in Kuruman (Figure 2b).

*Annotation 3* represents the religious significance of the place, particularly its link to Christianity. As noted above, the shelter is regularly used by individuals and groups to pray. Candles have been used to signify these actions, with the groupings of the candles being a metaphor for group prayer. The candles also refer to the themes of light and darkness, and are also placed to lead the way to the main part of the shelter (which is in fact how they are placed in reality), where the archaeological excavations have uncovered significant finds (Figure 2c).

*Annotation 4, 5, 6, and 7* places the viewer in front of the major archaeological finds at the site. The artefacts were digitally placed in their original context, but elevated above the ground, as coming from excavation area A. This addresses the challenges that Museums face when contextualising artefacts for the public. As noted above archaeology has a disreputable history, especially for African people. We wanted to reverse this narrative and create a celebration of our shared past. We, therefore, arranged the artefacts in such a way that they are neatly ordered, pleasing to view, and could be interpreted as a shrine to our ancestors, celebrating their significance. The fragments of ostrich eggshell were arranged into the shape of an ostrich egg, which visually tells of the story of their apparent original form, as storage containers (Figure 2d).

*Annotation 8* shows a fire near the centre of the main part of the North Shelter. People who use the site for cultural and religious purposes today often make fire in the shelter. Controlled fire is a uniquely human characteristic stretching far back in time. The presence of the fire, therefore, serves to link us back to our ancestors. The fire is animated to show movement and life in the 3D model (Figure 2e).

*Annotation 9* takes the viewer to the Tufa that was dated to show a wetter, more hospitable climate during the Middle Stone Age (Figure 2f).

*Annotation 10 *positions the viewer above the hill, affording a view over the landscape and a moment to absorb the totality of the hill. The text here describes the process of creating the 3D model of the hill, shelters, and objects, which provides essential contextual background information about the visualisation (Figure 2g).

*Annotation 11 and 12* position the viewer inside the South shelter. This shelter possesses rock art, created in more recent times, and represents a different people (herders) who inhabited the shelter in the Later Stone Age. The rock art is highlighted by offsetting a panel in the wall, which is given a higher resolution to the rest of the shelter (Figure 2h and i).

*Annotation 13* takes the view to the rock engravings. These engravings have been animated to enhance the viewers perception of their form. The engravings rise out of the rock as the enigmatic objects that they are (Figure 2j).

*Annotation 14* shows a simple image of the rock gong present at the site. Rock gongs are common features of the landscape Figure 2k).

## Discussion and Conclusions

This research has argued that producing 3D models of sites following the objective approach has emanated from a western cartesian methodology which has resulted in a focus on recording and visualising space, disregarding the intangibles of place. This unfortunately aligns with the colonial agenda which has sought to erode indigenous culture and identity in favour of colonial interests. To counter the objective approach to 3D documentation we provided a new approach that has as its focus the re-production of place, with the three aspects of agency, multi-vocality, and proximity forming its framework. This approach was practically implemented using our case study of Ga-Mohana Hill. While it can be argued that the methods (photogrammetry, model cleaning and optimisation) followed for the creation of the 3D model are almost identical (the differences being the formalised intention to be respectful during data capture, and informed choice of what should be recorded) to the objective approach it is the curation of the model, and the emphasis on co-production and multi-vocality, which saw the most change and affected the 3D visualisation that is presented to the public. The difference between the two approaches is in the shift away from (although not in detriment to) a focus creating perfect copies of space, to the end result which is representing that space as meaningful place to a public audience. This focus has been lacking in the 3D documentation community, but it is vital to the preservation of cultures as archaeological and living heritage sites enter the digital realm.

To establish agency in the 3D model a combination of visual and navigational devices was used with the aim of giving the model life. This was achieved by: the unpredictable (mind of its own) nature of the shadow when navigating; the presence of the Great Snake via its shadow moving across the wall which clearly announced its conceptual presence; animating the sparks of the fire, the waterfalls, engravings, and the rotating sign boards which presented the model as non-static and alive; showing the evidence of people at the place (candles, rock art, artefacts, fire, engravings) which re-humanised the model; and finally by allowing active engagement rather than passive presentation the model is able to create unique experience for all visitors, thereby creating new meaning, which is essential for the place to maintain its significance.

To achieve multi-vocality in the model, we adopted an ethical data capture policy that respects the site and the people who use it. We also aimed to represent the different significant uses of the place in a way that gave equal voice to all. This proved to be challenging in regards to the current uses as it became apparent that indigenous practices at Ga-Mohana are neglected and fragmented. From field work observations, people in the community, rather than being a homogenous group who are custodians of the site, are in fact cut-off from the site, not physically, but perhaps more problematically, culturally. Sites like these were places where moral and ethical values were imparted (Maape 2016), which as mentioned still happen to a lesser degree in the form of teenage initiation. The situation today is that generally most of the community no longer recognise the site as a place of value, and this could be partly a result of systematic cultural genocide by colonialism and apartheid. The community today face many social, economic, and increasingly, ecological problems. Sites like Ga-Mohana and the lessons attached to them, are precisely the kinds of wisdom that was destroyed under domination, including the fracturing of the community in various ways. Therefore, unlike communities where local indigenous groups advocate for the protection of a natural entity, and even perhaps fight for its rights, the situation here is that the community is no longer connected to sites of this nature, and the values attached to it. It is work being done by SM (Maape 2022), and other community members (The Workshop ko Kasi 2017), where advocacy, preservation, and defending the site is emerging, particularly from potential cultural destruction similar to the nearby archaeological site of Wonderwerk Cave, which has suffered damage from research activities. SM especially, aims to revive the intangible heritage of the site by including it and its stories in contemporary ecological challenges, both towards reversing the damage of apartheid and colonialism, as well as responding to climate change (see Kuramba in Maape (2022)).

Therefore, the aim of making the 3D model, and some of the decisions made regarding its representation, are influenced by this severing of people from their culture, and lack of advocacy for protecting the site. The place, in essence, belongs to all that use it, who as mentioned are a diverse group. So, an over emphasis on one representation of the site, has the potential of doing precisely what we believe is the risk, namely the exclusion of others. As a response to this, we have tried to include all the diverse elements, particularly the symbolic elements of the site that are present there, including the shadow of the snake, as an element that is not physically present like the symbols of the churches and the healers but is represented in the myths. From observing the new ways in which the youth are claiming the site for activities that address their current economic needs, we have proceeded to draw the site into new forms of engagement, and therefore, instead of creating complete secrecy and prohibition, are aiming to use this model as a way of creating new meaning, but specifically towards establishing contemporary forms of value and preservation. SM, who is a co-author of the 3D model, is exercising his rights as a member of this community, who has spent over 10 years advocating for the protection and reestablishment of the value of the site, to re-imagine the site in a digital form, representing as far as possible, the interests of the various groups mentioned above as perhaps a custodian of the site in both its physical and digital forms.

Proximity was established by creating a good quality digital re-production of the site using state-of-the-art photogrammetry capture and processing techniques with a focus to digitise the site as realistically and accurately as possible. To ensure the 3D model was inclusive and easily accessible meant we were forced to limit how much detail could be contained in the model due to file size limitations that were imposed to allow low-bandwidth and low-spec devices access. To mediate the experience the atmosphere of the site was recreated in the model using light and shadow. The website created to prepare viewers before entry into the model, and to provide contextualisation of the model, creates a sense of authority for the model, as museums do for artefacts. By providing information on how and who created the 3D model the chain of events leading back to the original is established, which along with the authority provided by the websites gives the model authenticity.

With the ever-advancing digital realm enveloping our lives and cultures, we are tending to view digital media as an important and necessary component and counterpart to physical things and places. We need to begin to shift our framing, understanding, and expectations of digitised entities from trivial digital copy, to valuable digital manifestation. We have shown that 3D models can offer a digital counterpart to a physical place and even offer a far richer experience, especially if the visitor is able to experience both the physical and digital manifestations of the place. We believe our approach is a step in the right direction to preserve and manifest the original potency of significant places in digital form, while at the same time creating decolonised and de-centred representations. Finally, we have shown that 3D models can also help with the preservation of indigenous cultures that are neglected, and we hope even revitalise them and for younger generations and help people re-connect with their past.

## Future Work

Following the current trajectory of the digital trend, future 3D digital replicas of places will exist as persistent places in the metaverse—an interconnected, embodied 3D digital virtual reality, currently being conceptualised, and touted as the new internet. Through the metaverse, we will be able to access cultural sites digitally in ever more realistic representations using immersive Mixed Reality technology (VR, AR, MR). This concept is alive today but is not yet fully implemented. To prepare for this world, future work should continuously question how archaeological and heritage sites are presented and engaged with to ensure their meaning, memory, and potency are transferred to an embodied internet.
